# Mental health effects caused by red imported fire ant attacks (*Solenopsis invicta*)

**DOI:** 10.1371/journal.pone.0199424

**Published:** 2018-06-25

**Authors:** Lei Wang, Yongyue Lu, Ripeng Li, Ling Zeng, Junling Du, Xiong Huang, Yijuan Xu

**Affiliations:** 1 Red imported Fire Ant Research Centre, South China Agricultural University, Guangzhou, China; 2 Department of Adult Psychiatric, Guangzhou Huiai Hospital, Affiliated Hospital of Guangzhou Medical University, Guangzhou, Guangdong, China; Leids Universitair Medisch Centrum, NETHERLANDS

## Abstract

Susceptible individuals who have suffered painful stings caused by red imported fire ants, *Solenopsis invicta*, usually experience physical health effects such as fever, dizziness, generalized urticaria, or other systemic reactions such as anaphylactic shock. Whether *S*. *invicta* stings also have negative effects on mental health is not clear. In the present study, the psychological impact of *S*. *invicta* stings was evaluated using a questionnaire that included a previously published posttraumatic stress disorder (PTSD) checklist, the Patient Health Questionnaire 9-item (PHQ-9), the Generalized Anxiety Disorder 7-item (GAD-7) scale, the Beck Anxiety Inventory (BAI), and the Pittsburgh Sleep Quality Index, 5th scale (PSQI(5)). A total of 96 valid questionnaires were obtained; 37 participants were placed in the stung group, and 59 participants were placed in the unstung group. Our results showed that symptoms of anxiety, depression and sleep disturbances were not associated with *S*. *invicta* stings (for GAD-7 scale, Pearson Chi-Square test, *χ*^2^ = 0.152, *df* = 1, *P* = 0.697; for the BAI, *χ*^2^ = 2.252, *df* = 1, *P* = 0.133; for the PHQ-9, *χ*^2^ = 0.098, *df* = 1, *P* = 0.754; for the PSQI(5), *χ*^2^ = 0.536, *df* = 1, *P* = 0.453). In total, 2 of 83 individuals stung by *S*. *invicta* met the criteria (>50) for PTSD. However, there was no significant difference on PTSD between those stung by *S*. *invicta* in the 30-day group and the over 30-day group (*χ*^2^ = 0.318, *df* = 1, *P* = 0.573). Overall, our data do not show an effect of *S*. *invicta* stings on mental health as measured using a range of indicators.

## Introduction

Arthropod bites and stings are common problems because arthropods are widely distributed, and during certain developmental stages, some arthropods must feed from other animals, including humans, whereas in other cases, biting or stinging devices are used for self-defense [1]. Arthropod bites and stings can have negative effects on human health. For example, wasp bites are painful. In Nepal, the death rate among hospitalized patients who were stung by wasps ranges from 5%-18% [2, 3]. Some diseases can be transmitted among humans through arthropod vector bites [4]. In addition to physical harm, arthropod bites and stings can also have negative effects on mental health [5–10]. Individuals exposed to bed bugs may experience sleep disturbances or develop symptoms of anxiety and post-traumatic stress disorder (PTSD), assessed by using standardized clinical mental health measures, i.e., the Patient Health Questionnaire 9-item (PHQ-9), Generalized Anxiety Disorder 7-item (GAD-7) scale, the Pittsburgh Sleep Quality Index 5th scale (PSQI(5)) and posttraumatic stress disorder (PTSD) checklist [6, 7].

The red imported fire ant, *Solenopsis invicta*, is a very aggressive species. Once a threat is perceived, fire ant workers quickly react en masse and attack any potential enemy to defend their colony [11]. Their stings are painful, likened to fire on the skin, and more dangerously, the venom from their stings contains alkaloids and proteins that cause skin redness, swelling, pustules, urticaria, edema, allergic shock, and even death [12, 13]. Given the widespread distribution of fire ants in human-inhabited areas, fire ant attacks are common. For instance, an investigation in China showed that 41% of investigated victims reported that they were stung by fire ants in city’s green belt, 32% of victims were stung in farmland, and 16% of victims were stung in parks [14]. In cities in the southeast region of the United States, almost 40% of the population may be stung by fire ants each year, and this proportion is even higher in rural areas [15]. In China, more than 30% of residents in fire ant-infested areas have been stung, and approximately 10% of these individuals experience fever and other symptoms including dizziness, generalized urticaria, or other systemic reactions including anaphylactic shock [16].

Unlike honey bees, a fire ant worker can sting repeatedly without dying [17]. Every people who is stung will experience sharp localized pain and itchiness for several days; scratching of the itchy areas predisposes the sufferers to secondary infections [12]. This painful experience is so pervasive that people living in the southern United States have a deeply emotional response to fire ant attacks [18]. In southern China, due to their frequent experience of fire ant stings when working in fields, many farm workers refuse to work in fire ant-infested fields, and some farmers have abandoned fields that became infested with fire ants [19]. Thus, we hypothesize that residents may become anxious and embarrassed after frequent *S*. *invicta* stings. To test this hypothesis, we investigated the mental health impact of *S*. *invicta* stings on residents. The objective of this study was to determine the psychological effects of *S*. *invicta* attacks using standardized clinical mental health measures.

## Materials and methods

### Data collection and measures

*S*. *invicta* are more active from spring to autumn [20], and most fire ant sting events in China occur in the same period [14] since that is when people are more likely to encounter them. Thus, we conducted our experiment during the warmer months of the year. We were invited to present educational seminars on the biology and management of *S*. *invicta* to residents in communities, farmers in suburbs and workers at gardening and landscaping companies in *S*. *invicta*-infested regions in Guangdong province several times, as we perform *S*. *invicta* investigation and management projects in Guangdong province. After the seminars, attendees were chosen at random to fill out an intervention health questionnaire. Meanwhile, participants were also recruited randomly through street investigations in Guangzhou city. Investigators were familiar with the symptoms of *S*. *invicta* stings and the biological characteristics of *S*. *invicta*. An intervention health questionnaire was considered acceptable if it was completed by the participant in its entirety. All of the surveys were reviewed by experienced physicians and entomologists in March and September of 2015.

Data were obtained through a questionnaire (Supplementary material, S1 Table). Symptoms of depression and anxiety were evaluated using the Brief Patient Health Questionnaire Mood Scale (PHQ-9) [21] and the Generalized Anxiety Disorder Screener 7-item (GAD-7) scale [22], which are based on the fifth editions of the Diagnostic and Statistical Manual of Mental Disorders (DSM-V) and the DSM-V-TR criteria [23], respectively. The Beck Anxiety Inventory (BAI) was also used [24]. Sleep disturbances were evaluated using questions 1–8 of the Pittsburgh Sleep Quality Index, 5th subscale (PSQI(5)) [25]. The Impact of Event Scale-Revised (IES-R) which includes intrusion, avoidance, and hyperarousal subscales [26], and a post-traumatic stress disorder (PTSD) checklist of symptoms based on criteria from the DSM-V were also used to survey the psychological conditions of people who had been stung by *S*. *invicta*. Other characteristics of participants were also collected including demographic features, histories of chronic medical and psychiatric conditions, exposure to other animals, and experiences with particularly stressful events within the previous year.

**Fig 1:**
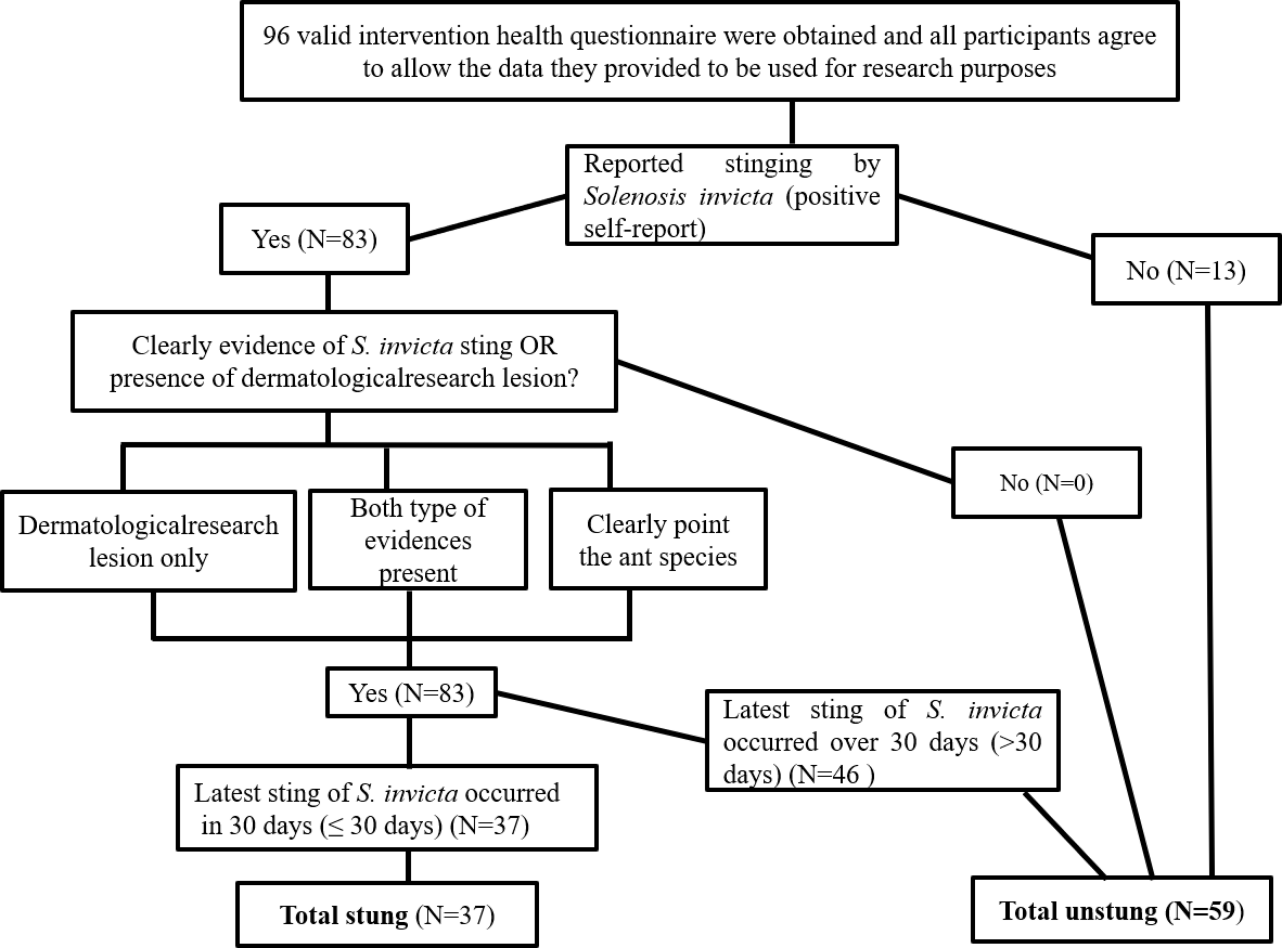
Algorithm for the attribution of a diagnosis of a *Solenopsis invicta* sting to the presence of characteristic lesions and clear evidence.

*S*. *invicta* sting statuses were initially determined by self-reports. Details related to *S*. *invicta* stings were recorded, such as dermatological lesions and ant samples (Fig 1). Participants who self-reported attacks by *S*. *invicta* were asked to identify the culprit insect on an identification tool containing pictures of *S*. *invicta* and other aggressive insects such as wasps, bees, and other ants. Meanwhile, investigators also confiremed whether participants experienced symptoms associated with *S*. *invicta* stings such as dermatological evidence or allergic reactions after attacks by the insects (Fig 1).

Individuals who were stung by *S*. *invicta* within 30 or fewer days were placed in the stung group. Individuals who had not experienced an *S*. *invicta* stung or who had been stung by *S*. *invicta* in the past but not within the previous 30 days were placed in the un-stung group. Although fire ant stings are painful and the venom can cause skin redness, swelling, pustules, urticaria, edema and allergic shock, the symptoms do not last for 30 days, and nearly disappear 2–3 weeks after the sting (personal observation). Meanwhile, we also referred to the research of Susser et al. [6], who evaluated whether or not bed bug infestations can cause mental health effects. In their study, 30 days was used as the time period for determining if subjects were assigned to the exposed or unexposed group [6]. Unlike bed bugs, *S*. *invicta* is not a hematophagous insect, and its stings are also easier to avoid than bites from bed bugs. Taken together, we believe that the use of 30 days as a cut off for classifying individuals as being stung or unstung is justified.

Since “symptoms last for more than 1 month” is a required criterion in the DSM-V criteria for PTSD [23], we compared the intrusion, avoidance, and hyperarousal subscales of the PTSD checklist between the stung and unstung groups to evaluate whether fire ant stings can cause PTSD.

A total of 96 valid questionnaires were obtained, out of which 37 participants were classified as stung and 59 participants were classified as unstung (Fig 1). We further analyzed the mental health effects of *S*. *invicta* attacks in mainland China based on these data.

### Ethics, consent and permissions

This study was reviewed and approved by The Medical Ethics Committee of Guangzhou Huiai Hospital (Supplementary material, S1 Fig and S1 File). All of the participants in this investigation agreed to allow the data they provided to be used for research purposes (Supplementary material, S2 File).

### Statistical analysis

A χ^2^ analysis was used to compare the different characteristics between the stung and unstung groups. Scores for the PHQ-9, GAD-7 scale, BAI and PSQI(5) were classified as “present” or “absent”. Symptoms were considered “present” when scores reached 10 or higher out of 27 possible points on the PHQ-9, five or higher out of 21 on the GAD screen, 45 or higher of 74 on the BAI, and 10 or higher of 24 on the PSQI(5) [6, 24]. Scores on the BAI were corrected using the formula y = int(x) before the test, where y represents the scores we used in the test and x represents the original scores in the investigation [24].

The IES-R was only used to evaluate the psychiatric conditions of people who were stung by *S*. *invicta*. There were two groups in this test: 30-day group (individuals who were stung by *S*. *invicta* within in the previous 30 days, i.e., the stung group) and the over 30-day group (individuals from the unstung group who were stung by *S*. *invicta* in the past but not within the previous 30 days). Scores of 35 above were suggestive of PTSD [27]. The IES-R has three parts; each part was scored and the total scores were calculated. All of the statistical data from the IES-R were tested for normal distribution using the Shapiro-Wilk test and for the homogeneity of variances using Levene’s test. The independent t-test was used to test the difference between those stung by *S*. *invicta* in the 30-day group and the over 30-day group.

The internal consistency and reliability of the psychometric tools (PHQ-9, GAD-7, PSQI(5), BAI, and IES-R) were determined by computing Cronbach’s alpha values [28]. In general, items with an alpha correlation of 0.7 are considered to have adequate internal consistency [29].

Analyses were performed using SPSS 18.0 (SPSS Inc., Chicago, IL, United States).

## Results

A total of 96 valid questionnaires were obtained in the investigation, with 37 participants classified as stung and 59 participants classified as unstung as described in the Materials and methods section (Fig 1). There were no significant differences between stung and unstung individuals on the characteristics shown in Table 1 except for “Education” (Pearson Chi-Square test, *χ*^2^ = 8.339, *df* = 1, *P* = 0.004).

**Table 1:** Characteristics and instrument scores of participants according to *Solenopsis invicta* sting status.

Characteristics	Total number of participants	Number of individuals in stung group (percentage*)	Number of individuals in unstung group (percentage*)	*χ*^2^	*df*	*P*
Sex				0.355	1	0.551
Male	68	28 (41.18%)	40 (58.82%)			
Female	28	9 (32.14%)	19 (67.86%)			
Age (Years)				1.428	1	0.232
≤30	45	14 (31.11%)	31 (68.89%)			
≥31	51	23 (45.10%)	28 (54.90%)			
Legally married				0.704	1	0.402
Yes	48	21 (43.75%)	27 (56.25%)			
No	48	16 (33.33%)	32 (66.67%)			
Education†				8.339	1	0.004
High school or less	29	18 (62.07%)	11 (37.93%)			
More than high school	67	19 (28.36%)	48 (71.64%)			
Employment status				0.271	1	0.603
Employed	79	29 (36.71%)	50 (63.29%)			
Unemployed	17	8 (47.06%)	9 (52.94%)			
Disturbed by other insects				0.366	1	0.545
Yes	24	11 (45.83%)	13 (54.17%)			
No	72	26 (36.11%)	46 (63.89%)			
Medical diagnosis				2.328	1	0.127
Yes	13	8 (61.54%)	5 (38.46%)			
No	83	29 (34.94%)	54 (65.06%)			
Stressful event in last year				0.375	1	0.541
Yes	34	15 (44.12%)	19 (55.88%)			
No	62	22 (35.48%)	40 (64.52%)			
Anxiety symptoms (GAD-7)				0.152	1	0.697
Present	40	14 (35.00%)	26 (65.00%)			
Absent	56	23 (41.07%)	33 (58.93%)			
BAI				2.252	1	0.133
Present	16	3 (18.75%)	13 (81.25%)			
Absent	80	34 (42.50%)	46 (57.50%)			
Depressive symptoms (PHQ-9)				0.098	1	0.754
Present	13	4 (30.77%)	9 (69.23%)			
Absent	83	33 (39.76%)	50 (60.24%)			
Sleep disturbances (PSQI(5))				0.563	1	0.453
Present	16	8 (50.00%)	8 (50.00%)			
Absent	80	29 (36.25%)	51 (63.75%)			
Total	96					

*Percentage was calculated by (number of individuals in stung or unstung group/ total number of participants) for each index.

†This variable was significantly different for the *S*. *invicta* unstung group in the Pearson χ^2^ analysis (*P* < 0.05), two-sided test.

Our results showed that Pearson χ^2^ scores for the univariate associations between *S*. *invicta* sting status and the dependent variables were not significant at *P*<0.05 for GAD-7 (*χ*^2^ = 0.152, *df* = 1, *P* = 0.697), the BAI (*χ*^2^ = 2.252, *df* = 1, *P* = 0.133), the PHQ-9 (*χ*^2^ = 0.098, *df* = 1, *P* = 0.754), and PSQI(5) (*χ*^2^ = 0.536, *df* = 1, *P* = 0.453) (Table 1).

According to the recommended scoring rubric for the PTSD checklist, the scores in the 30-day group ranged from 0–32 with a mean of 12.622 and SE of 1.449, while in the over 30-day group, the scores ranged from 2–62 with a mean of 14.435 and SE of 1.730. None of the 37 stung individuals met the criteria (>50) for PTSD after experiencing an *S*. *invicta* sting in the 30-day group, but 2 of the 46 stung individuals met the criteria for PTSD in the over 30-day group. However, there was no significant difference between the 30-day and over 30-day groups (*χ*^2^ = 0.318, *df* = 1, *P* = 0.573). Further data analyses showed that there were no significant differences in the intrusion, avoidance, or hyperarousal subscales of the PTSD checklist between the two groups (Table 2).

**Table 2:** Scores on subscales of the PTSD checklist at different time periods after the most recent *Solenopsis invicta* sting.

Subscale	Period after the latest sting		*t*	df	*P*
	≤30	>30			
N*	37	46			
Intrusion	4.351±0.552	5.174±0.641	-0.946	81	0.347
Avoidance	4.838±0.816	5.174±0.780	-0.296	81	0.768
Hyperarousal	3.432±0.404	4.087±0.516	-0.962	81	0.339
Total	12.622±1.449	14.435±1.730	0.291	81	0.438

*N indicates the number of *S*. *invicta* sting victims.

The Cronbach’s alpha values calculated from the data for the PHQ-9, GAD-7, BAI, PSQI(5), and IES-R were found to be 0.834, 0.893, 0.763, 0.928, and 0.918, respectively.

## Discussion

Our study showed that, based on the DSM-V criteria, depression and sleep disturbances did not occur among individuals who were stung by *S*. *invicta*. However, 2 of 83 individuals who were stung met the criteria (>50) for PTSD.

To the best of our knowledge, this study is the first to evaluate the association between *S*. *invicta* attacks and symptoms of anxiety, depression, sleep disturbances, and PTSD. Previous studies showed that infestations of biting arthropods, such as bed bugs, pigeon fleas, and head lice, can cause negative effects on mental health [5–10]. However, our study suggested that *S*. *invicta* attacks were not associated with measurable depression or sleep disturbance symptoms. Unlike bed bugs, pigeon fleas, and head lice, *S*. *invicta* is not a hematophagous insect that lives with and bothers individuals daily, especially at night. *S*. *invicta* stings are also easier to avoid than bites from bed bugs, pigeon fleas, and head lice. These differences may be why *S*. *invicta* stings did not cause anxiety, depression or sleep disturbance symptoms.

Our findings indicated that some individuals may develop PTSD after receiving an *S*. *invicta* sting. A similar phenomenon has also been reported in bed bug infestations; one in 135 people who experienced bites during infestations met the criteria (>50) for PTSD [7]. One hundred and five cases of fire ant stings in China were obtained from the internet from 2003 to 2015, and Zhao and Xu [14] found that all of the victims experienced reactions after the stings. Of those stung, 60 (57.14%) experienced itchiness, 57 (54.28%) had redness at presentation, 14 (13.33%) presented in shock, and one died [14]. This experience among certain individuals may meet the DSM-V Criterion A for an “actual or threatened death or serious physical injury” and may also explain why some farmers who experience *S*. *invicta* stings refuse to return to work in *S*. *invicta*-infested fields. PTSD is classified into three types based on DSM-V criteria: acute (in which the clinical course lasts less than one month), chronic (in which the clinical course lasts more than three months), and delayed (in which PTSD develops 1–3 month after the traumatic event) [23]. Our results showed that 2 of 46 individuals meet criteria for developing PTSD symptoms 30 days after experiencing an *S*. *invicta* sting. We hypothesize that the resulting type of PTSD would be the chronic or delayed form. Further or accompanying research should involve thorough case reports of individuals who meet the criteria for PTSD following an *S*. *invicta* sting. Further investigation would enable us to understand if *S*. *invicta* sting can lead to a true PTSD diagnosis and to understand why and in which circumstances individuals may develop PTSD.

## Supporting information

S1 Fig Exemption determination request form-in Chinese. (JPG)

S1 File Exemption determination request Form-English version. (DOCX)

S2 File The human participant consent form-in Chinese. (DOCX)

S1 Table Questionnaire. (DOCX)
