# Supplementary figures and images for: Mental health effects caused by red imported fire ant attacks (Solenopsis invicta)
Source: PLoS One. 2018 Jun 25;13(6):e0199424. doi: 10.1371/journal.pone.0199424 (PMC6016926; doi:10.1371/journal.pone.0199424)

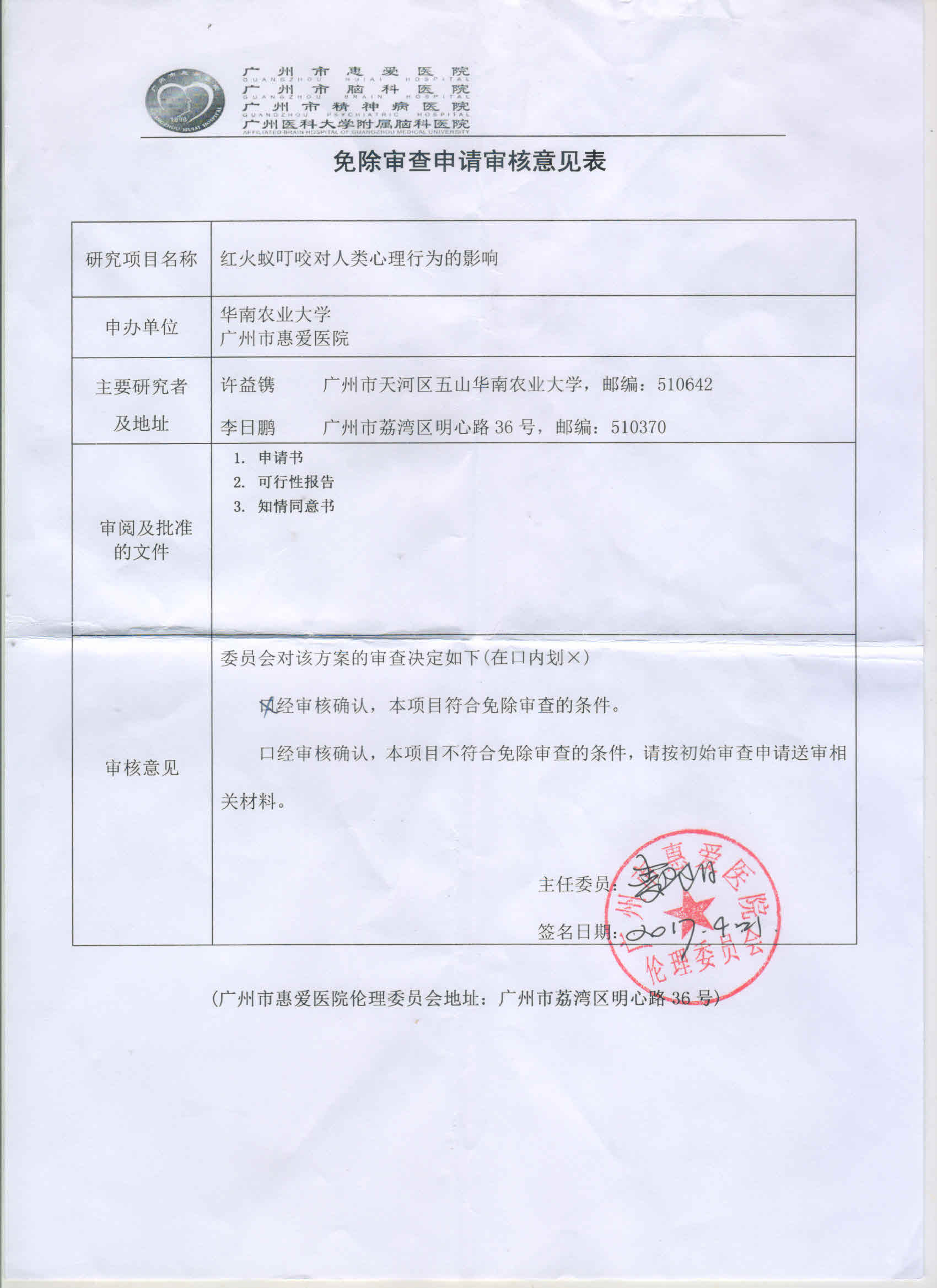

Supplement: S1 Fig — (JPG) [file pone.0199424.s001.jpg]
