# Supplementary material for: Mental health effects caused by red imported fire ant attacks (Solenopsis invicta)
Source: PLoS One. 2018 Jun 25;13(6):e0199424. doi: 10.1371/journal.pone.0199424 (PMC6016926; doi:10.1371/journal.pone.0199424)
Supplement: S1 File — (DOCX) [file pone.0199424.s002.docx]

Exempting Determination Request Form

| Project | Mental Health Effects Caused By Red Imported Fire Ant Attacks |
| --- | --- |
| Research Institution | South China Agricultural University  Guangzhou Huiai Hospital |
| Main Researchers and Their Address | Xuyijuan, 483 Wushan Road, Tianhe District, Guangzhou, Guangdong, China  Li Ripeng, 36 Mingxin Road, Liwan District, Guangzhou, Guangdong, China |
| Document provided by applicants | 1. the proposal  2. the feasibility report  3. the informed consent |
| Comments of the Medical Ethics Committee | - **√** After reviewed the documents provide by applicants, the Medical Ethics Committee confirmed that the ethics approval was not needed. - After reviewed the documents provide by applicants, the Medical Ethics Committee confirmed that the ethics approval was needed.   Chairman: Mai Weiyang  The Medical Ethics Committee of Guangzhou Huiai Hospital |

Address of the Committee on Medical Ethics Guangzhou Huiai Hospital: 36 Mingxin Road, Liwan District, Guangzhou, Guangdong, China
