# Supplementary material for: Mental health effects caused by red imported fire ant attacks (Solenopsis invicta)
Source: PLoS One. 2018 Jun 25;13(6):e0199424. doi: 10.1371/journal.pone.0199424 (PMC6016926; doi:10.1371/journal.pone.0199424)
Supplement: S2 File — (DOCX) [file pone.0199424.s003.docx]

**“红火蚁叮咬对人类心理影响”研究的知情同意书**

我们将开展一项“红火蚁叮咬对人类心理影响”的研究，您的具体情况符合该研究的入组条件，因此，我们想邀请您参加这项研究。

本知情同意书将向您介绍该研究的目的、方法和您的获益、风险、不便以及权益等，请仔细阅读后慎重做出是否参加研究的决定。当研究者向您说明和讨论知情同意书时，您可以随时提问并请他/她解释您不明白的地方。您可以与家人、朋友等讨论后在做决定。

**一、研究背景及研究目的**

红火蚁*Solenopsis invicta*是一种危险性的入侵生物，其习性凶猛，具有主动攻击性。在其受到侵扰后，工蚁会从蚁巢内蜂拥而出寻找入侵者。首先使用上鄂叮咬住入侵者，然后用尾部的蛰针刺进入侵者体内注射含有生物碱和毒蛋白的毒液。被红火蚁叮咬后，所有的人都会有痒痛反应，大多数人会出现红肿、伤口化脓等症状，少部分人会出现发烧、暂时性失明、呕吐、荨麻疹、休克甚至是死亡等严重的过敏反应，而且伤口还容易引起细菌性的二次感染。那么红火蚁的叮蛰是否会引起被侵扰人群产生心理疾病？我们拟通过心理调查问卷对红火蚁发生区人群开展调查，以深入了解红火蚁侵扰对人类心理健康的影响。

**二、参研人数**

本研究计划招募100名受试者。

**三、研究方法**

1. 研究对象

1) 入组标准

A. 被红火蚁叮咬者；

B. 年龄在18-55 岁之间；

C. 经过本人知情同意。

2) 排除标准

A. 不符合入组标准；

B. 伴有严重躯体疾病、脑器质性疾病等；

C. 排除精神分裂症、重症抑郁、双相障碍者、躁狂和分裂症后抑郁等DSM-IV 轴I诊断的患者。

3) 样本来源和样本含量

A. 样本来源：广州市人群；

B. 样本含量：96例。

4) 正常研究对象：59例

A. 无任何躯体、精神疾病病史，从无任何DSM-IV轴I诊断；

B. 样本来源为随机抽查人群；

2. 量表评定

1) PHQ-9：是基于DSM-IV的诊断标准而修订的关于抑郁的一个筛查表。每个条目的分值设置为0-3分，共有9个条目总分值27分。根据分值评估抑郁程度。

2) GAD-7：可用于广泛性焦虑的筛查及症状严重度的评估，是PHQ的一个组成部分。广泛性焦虑量表由7个项目组成，每个条目的分值设置为0-3分，总分范围0-21分，根据得分评估焦虑程度。

3) BAI：含有21个自评项目，分4级评分（“1”表示无；“2”表示轻度，无多大烦恼；“3”表示中度，感到不适但尚能忍受；“4”表示重度，只能勉强忍受），可以评定受试者被多种焦虑症状烦扰的程度。

4）PSQI：PSQI用于评定被试最近1个月的睡眠质量，由9个自评和5个他评条目构成，而其中18个条目组成7个因子，每个因子按0-3分等级计分，累积各因子成分得分为匹茨堡睡眠质量指数量表的总分，总分范围为0-21，得分越高，表示睡眠质量越差。

5）ISE-R：生活事件冲击量表是一个对被试对特殊的生活事件的灾难性体验的进行测量和评估的自陈式问卷。ISE-R共有22题，分为侵袭性症状，回避症状，高唤醒症状三个分量表。α系数：Weiss and Marmar (1997)报告三个分量表的内在一致性信度很高（α从 .87 到 .92）。

**四、风险和不适**

问卷调查将占用您大约25分钟的时间，调查问卷中的某些问题可能会让您感到不舒服，您可以拒绝回答。

**五、关于费用**

本研究不提供其他补偿或报酬。

**六、参加本项目可获得的益处**

您的参与将有助于研究者得到更多可靠的研究数据，有益于更加深入理解红火蚁对人类健康的影响。

**七、保密问题**

您参加研究及在研究中的个人资料均属保密。必要时，政府管理部门或伦理委员会的成员按规定可以在研究单位查阅您的个人信息。这项研究结果发表时，将不会披露您的任何身份信息。

**八、参加和退出项目的权利**

您是否参加这个研究完全是自愿的。如果您不愿意，可以拒绝参加。即使您同意参加以后，您也可以在任何时间改变主意，告诉研究者退出研究。

**九、联系方式**

如果您有与本研究相关的任何问题，请联系研究者。研究者姓名许益镌，联系电话020-85283518。如果您有与自身权益相关的问题，可与广州惠爱医院医学伦理委员会联系，联系电话：020-81894502。

**请研究者和受试者仔细阅读相关声明并签名。**

**研究者声明**

“我已告知该受试者’红火蚁叮咬对人类心理影响’的研究背景、目的、步骤、风险及获益情况，给予他/她足够的时间阅读知情同意书、与他人讨论，并解答了其有关研究的问题；我已告知该受试者当遇到与研究相关的问题时可随时与项目负责人许益镌博士联系，遇到与自身权利/权益相关问题时随时与广州惠爱医院医学伦理委员会联系，并提供了准确的联系方式，已告知该受试者他/她可以无需任何理由退出本研究，我已告知该受试者他/她将得到这份知情同意书的副本，上面包含我和他/她的签名。”

获得知情同意的研究者签名 日期

**受试者声明**

“我已被告知’红火蚁叮咬对人类心理影响’的研究背景、目的、步骤、风险及获益情况。我有足够的时间和机会进行提问，我对问题的答复很满意。我也被告知，当我有问题、想反映困难、顾虑、对研究有建议，或想进一步获得信息，或为研究提供帮助时，应当联系谁。我已经阅读这份知情同意书，并且同意参加本研究。我知道我可以在研究期间任何时候无需任何理由推出本研究。我被告知我将得到这份知情同意书的副本，上面包含我和研究者的签名。”

受试者签名 日期
