# Supplementary material for: Mental health effects caused by red imported fire ant attacks (Solenopsis invicta)
Source: PLoS One. 2018 Jun 25;13(6):e0199424. doi: 10.1371/journal.pone.0199424 (PMC6016926; doi:10.1371/journal.pone.0199424)
Supplement: S1 Table — (DOCX) [file pone.0199424.s004.docx]

**Questionnaire on Psychological Effects of *Solenopsis invicta***

| Date |  | | Investigation Site | | | | |  | | | | | | | Sex | | | Male □ Female □ |
| --- | --- | --- | --- | --- | --- | --- | --- | --- | --- | --- | --- | --- | --- | --- | --- | --- | --- | --- |
| Age | ≤30 □ 31-40 □ 41~50 □ >50 □ | | | | | | | | | | | | | Legally married | | | Yes □ No □ | |
| Education | Less than high school □ High school or more □ | | | | | | | | | | Employment | | | | Employed □ Unemployed □ | | | |
| Number of family member | | 1 □ 2 □ ≥3□ | | Period of last attack of *S. invicta* | | | | | | | | | | | | ≤30 days □ >30 days □ | | |
| How often have you been bothered by fire ant in 30 days | | | | | | | | | | None □ ≤ 2 times/week □ ≥3 times/week □ | | | | | | | | |
| Have you been bothered by other animals in 30 days | | | | | | | Yes □ No □ | | | | | | | | | | | |
| How often have you been bothered by other animals | | | | | | | None □ ≤ 2 times/week □ ≥3 times/week □ | | | | | | | | | | | |
| Perceived sufficient means | | | | | | | Yes □ No □ | | | | | | | | | | | |
| History of chronic medical (>6 months induration) | | | | | Yes □ No □ | | | | | | | | | | | | | |
| History of psychiatric medical (>6 months induration) | | | | | | | | | | Yes □ No □ | | | | | | | | |
| Stressful event in last year | | | | | | | | | | | | Yes □ No □ | | | | | | |
| **First section: Over the last 2 weeks, how often have you been bothered by any of the following** | | | | | | | | | | | | | | | | | | |
| 1. Little interest or pleasure in doing things? | | | | | | | | | | Not at all □ Several days □ More than half the days □ Nearly every day □ | | | | | | | | |
| 1. Feeling down, depressed, or hopeless? | | | | | | | | | | Not at all □ Several days □ More than half the days □ Nearly every day □ | | | | | | | | |
| 1. Trouble falling or staying asleep, or sleeping too much? | | | | | | | | | | Not at all □ Several days □ More than half the days □ Nearly every day □ | | | | | | | | |
| 1. Feeling tired or having little energy? | | | | | | | | | | Not at all □ Several days □ More than half the days □ Nearly every day □ | | | | | | | | |
| 1. Poor appetite or overeating? | | | | | | | | | | Not at all □ Several days □ More than half the days □ Nearly every day □ | | | | | | | | |
| 1. Feeling bad about yourself-or that you are a failure or have let yourself or your family down? | | | | | | | | | | Not at all □ Several days □ More than half the days □ Nearly every day □ | | | | | | | | |
| 1. Trouble concentrating on things, such as reading the newspaper or watching television? | | | | | | | | | | Not at all □ Several days □ More than half the days □ Nearly every day □ | | | | | | | | |
| 1. Moving or speaking so slowly that other people could have noticed? Or the opposite-being so fidgety or restless that you have been moving around a lot more than usual? | | | | | | | | | | Not at all □ Several days □ More than half the days □ Nearly every day □ | | | | | | | | |
| 1. Thoughts that you would be better off dead or of hurting yourself in some way? | | | | | | | | | | Not at all □ Several days □ More than half the days □ Nearly every day □ | | | | | | | | |
| **Second section: Over the last 2 weeks, how often have you been bothered by any of the following** | | | | | | | | | | | | | | | | | | |
| 1. Feeling nervous, anxious or on edge | | | | | | | | | | | | | Not at all □ Several days □ More than half the days □ Nearly every day □ | | | | | |
| 1. Not being able to stop or control worrying | | | | | | | | | | | | | Not at all □ Several days □ More than half the days □ Nearly every day □ | | | | | |
| 1. Worrying too much about different things | | | | | | | | | | | | | Not at all □ Several days □ More than half the days □ Nearly every day □ | | | | | |
| 1. Trouble relaxing | | | | | | | | | | | | | Not at all □ Several days □ More than half the days □ Nearly every day □ | | | | | |
| 1. Being so restless that it is hard to sit still | | | | | | | | | | | | | Not at all □ Several days □ More than half the days □ Nearly every day □ | | | | | |
| 1. Becoming easily annoyed or irritable | | | | | | | | | | | | | Not at all □ Several days □ More than half the days □ Nearly every day □ | | | | | |
| 1. Feeling afraid as if something awful might happen | | | | | | | | | | | | | Not at all □ Several days □ More than half the days □ Nearly every day □ | | | | | |
| **Third section: Over the last 2 weeks, how often have you been bothered by any of the following** | | | | | | | | | | | | | | | | | | |
| 1. Cannot get to sleep within 30 minutes | | | | | | | | | Not during the past month □ Less than once a week □  Once or twice a week □ Three or more times a week □ | | | | | | | | | |
| 1. Wake up in the middle of the night or early morning | | | | | | | | | Not during the past month □ Less than once a week □  Once or twice a week □ Three or more times a week □ | | | | | | | | | |
| 1. Have to get up to use the bathroom | | | | | | | | | Not during the past month □ Less than once a week □  Once or twice a week □ Three or more times a week □ | | | | | | | | | |
| 1. Cannot breathe comfortablely | | | | | | | | | Not during the past month □ Less than once a week □  Once or twice a week □ Three or more times a week □ | | | | | | | | | |
| 1. Cough or snore loudly | | | | | | | | | Not during the past month □ Less than once a week □  Once or twice a week □ Three or more times a week □ | | | | | | | | | |
| 1. Feel too cold | | | | | | | | | Not during the past month □ Less than once a week □  Once or twice a week □ Three or more times a week □ | | | | | | | | | |
| 1. Feel too hot | | | | | | | | | Not during the past month □ Less than once a week □  Once or twice a week □ Three or more times a week □ | | | | | | | | | |
| 1. Had bad dream | | | | | | | | | Not during the past month □ Less than once a week □  Once or twice a week □ Three or more times a week □ | | | | | | | | | |
| **Fourth section: Over the last 2 weeks, how often have you been bothered by any of the following** | | | | | | | | | | | | | | | | | | |
| 1. Numbness or tingling | | | | | | | | | Not at all □ Mild □ Moderate □ Severely □ | | | | | | | | | |
| 1. Feeling hot | | | | | | | | | Not at all □ Mild □ Moderate □ Severely □ | | | | | | | | | |
| 1. Wobbliness in legs | | | | | | | | | Not at all □ Mild □ Moderate □ Severely □ | | | | | | | | | |
| 1. Unable to relax | | | | | | | | | Not at all □ Mild □ Moderate □ Severely □ | | | | | | | | | |
| 1. Fear of the worst happening | | | | | | | | | Not at all □ Mild □ Moderate □ Severely □ | | | | | | | | | |
| 1. Dizzy or lightheaded | | | | | | | | | Not at all □ Mild □ Moderate □ Severely □ | | | | | | | | | |
| 1. Heart pounding or racing | | | | | | | | | Not at all □ Mild □ Moderate □ Severely □ | | | | | | | | | |
| 1. Unsteady | | | | | | | | | Not at all □ Mild □ Moderate □ Severely □ | | | | | | | | | |
| 1. Terrified | | | | | | | | | Not at all □ Mild □ Moderate □ Severely □ | | | | | | | | | |
| 1. Nervous | | | | | | | | | Not at all □ Mild □ Moderate □ Severely □ | | | | | | | | | |
| 1. Feelings of choking | | | | | | | | | Not at all □ Mild □ Moderate □ Severely □ | | | | | | | | | |
| 1. Hands trembling | | | | | | | | | Not at all □ Mild □ Moderate □ Severely □ | | | | | | | | | |
| 1. Shaky | | | | | | | | | Not at all □ Mild □ Moderate □ Severely □ | | | | | | | | | |
| 1. Fear of losing control | | | | | | | | | Not at all □ Mild □ Moderate □ Severely □ | | | | | | | | | |
| 1. Difficulty breathing | | | | | | | | | Not at all □ Mild □ Moderate □ Severely □ | | | | | | | | | |
| 1. Fear of dying | | | | | | | | | Not at all □ Mild □ Moderate □ Severely □ | | | | | | | | | |
| 1. Scared | | | | | | | | | Not at all □ Mild □ Moderate □ Severely □ | | | | | | | | | |
| 1. Indigestion or discomfort in abdomen | | | | | | | | | Not at all □ Mild □ Moderate □ Severely □ | | | | | | | | | |
| 1. Faint | | | | | | | | | Not at all □ Mild □ Moderate □ Severely □ | | | | | | | | | |
| 1. Face flushed | | | | | | | | | Not at all □ Mild □ Moderate □ Severely □ | | | | | | | | | |
| 1. Sweating (not due to heat) | | | | | | | | | Not at all □ Mild □ Moderate □ Severely □ | | | | | | | | | |
| **Fifth section: Over the last 2 weeks, how often have you been bothered by any of the following** | | | | | | | | | | | | | | | | | | |
| 1. Any reminders brought back feelings about it | | | | | | None □ Rarely□ Sometimes□ Often□ Always□ | | | | | | | | | | | | |
| 1. I had trouble staying asleep | | | | | | None □ Rarely□ Sometimes□ Often□ Always□ | | | | | | | | | | | | |
| 1. Other things kept making me think about it | | | | | | None □ Rarely□ Sometimes□ Often□ Always□ | | | | | | | | | | | | |
| 1. I felt irritable and angry | | | | | | None □ Rarely□ Sometimes□ Often□ Always□ | | | | | | | | | | | | |
| 1. I avoided letting myself get upset... | | | | | | None □ Rarely□ Sometimes□ Often□ Always□ | | | | | | | | | | | | |
| 1. I thought about it when I didn’t mean to | | | | | | None □ Rarely□ Sometimes□ Often□ Always□ | | | | | | | | | | | | |
| 1. I felt as if it hadn’t happened or wasn’t real | | | | | | None □ Rarely□ Sometimes□ Often□ Always□ | | | | | | | | | | | | |
| 1. I stayed away from reminders about it | | | | | | None □ Rarely□ Sometimes□ Often□ Always□ | | | | | | | | | | | | |
| 1. Pictures about it popped into my mind | | | | | | None □ Rarely□ Sometimes□ Often□ Always□ | | | | | | | | | | | | |
| 1. I was jumpy and easily startled | | | | | | None □ Rarely□ Sometimes□ Often□ Always□ | | | | | | | | | | | | |
| 1. I tried not to think about it | | | | | | None □ Rarely□ Sometimes□ Often□ Always□ | | | | | | | | | | | | |
| 1. I was aware that I still had a lot of feelings... | | | | | | None □ Rarely□ Sometimes□ Often□ Always□ | | | | | | | | | | | | |
| 1. My feelings about it were kind of numb | | | | | | None □ Rarely□ Sometimes□ Often□ Always□ | | | | | | | | | | | | |
| 1. I found myself acting or feeling like I was back... | | | | | | None □ Rarely□ Sometimes□ Often□ Always□ | | | | | | | | | | | | |
| 1. I had trouble falling asleep | | | | | | None □ Rarely□ Sometimes□ Often□ Always□ | | | | | | | | | | | | |
| 1. I had waves of strong feelings about it | | | | | | None □ Rarely□ Sometimes□ Often□ Always□ | | | | | | | | | | | | |
| 1. I tried to remove it from my memory | | | | | | None □ Rarely□ Sometimes□ Often□ Always□ | | | | | | | | | | | | |
| 1. I had trouble concentrating | | | | | | None □ Rarely□ Sometimes□ Often□ Always□ | | | | | | | | | | | | |
| 1. Reminders caused me to have physical reactions... | | | | | | None □ Rarely□ Sometimes□ Often□ Always□ | | | | | | | | | | | | |
| 1. I had dreams about it | | | | | | None □ Rarely□ Sometimes□ Often□ Always□ | | | | | | | | | | | | |
| 1. I felt watchful and on guard | | | | | | None □ Rarely□ Sometimes□ Often□ Always□ | | | | | | | | | | | | |
| 1. I tried not to talk about it | | | | | | None □ Rarely□ Sometimes□ Often□ Always□ | | | | | | | | | | | | |
